# Semaglutide promotes the proliferation and osteogenic differentiation of bone-derived mesenchymal stem cells through activation of the Wnt/LRP5/β-catenin signaling pathway

**DOI:** 10.3389/fphar.2025.1539411

**Published:** 2025-03-10

**Authors:** Yawei Tian, Huiming Liu, Xiaoxue Bao, Yukun Li

**Affiliations:** ^1^ Department of Endocrinology, Hebei Medical University Third Hospital, Shijiazhuang, China; ^2^ Department of Stomatology, Hebei Medical University Second Hospital, Shijiazhuang, China

**Keywords:** GLP-1, semaglutide, osteogenic differentiation, LRP5, signaling pathway

## Abstract

Diabetes mellitus is a global disease in which alterations in the internal environment disrupt the bone-fat balance, contributing to osteoporosis. Semaglutide, a single-target, long-acting glucagon-like peptide-1 receptor agonist (GLP-1RA), has been shown to promote osteogenesis *in vitro*, but the underlying mechanism remains unclear. In this study, the ability of Semaglutide to promote the proliferation of bone-derived mesenchymal stem cells (BMSCs) was determined by CCK-8 kit and flow cytometry, Alkaline phosphatase (ALP) staining and alizarin red S staining showed that semaglutide increased ALP activity and the proportion of mineralised nodules during induction of osteogenesis, wound healing assay to evaluate the pro-migratory ability of semaglutide on BMSCs.Western blotting and RT-PCR showed that semaglutide promoted the mRNA and protein expression of osteocalcin (OCN) and Runt-related transcription factor 2 (RUNX2), and further determined the OCN expression level by immunofluorescence. RNA sequencing was performed to analyze the mechanisms underlying BMSC osteogenesis after semaglutide intervention. Enrichment of RNA sequencing data indicated that the Wnt/LRP5/β-catenin pathway was activated after treatment with semaglutide. Western blotting further confirmed the upregulation of Wnt pathway-associated protein levels by semaglutide. Dickkopf-1 (DKK1) and LiCl (lithium chloride) are common inhibitors and agonists of the Wnt/β-catenin pathway. The addition of semaglutide resulted in the partial reversal of the inhibitory effect of DKK1 on osteogenic differentiation, with the administration of LiCl and semaglutide further accelerating the osteogenic process. In addition to alterations in gene and protein expression levels, these changes are also reflected in alkaline phosphatase (ALP) activity and calcium deposition. Therefore, we suggest that semaglutide can promote the proliferation and osteogenic differentiation of BMSCs *in vitro* via the Wnt/LRP5/β-catenin signalling pathway.

## 1 Introduction

Osteoporosis (OP) is an age-related condition characterized by systemic skeletal involvement, low bone volume, and destruction of bone microstructure (Ensrud and Crandall, 2024). A T-score ≤ −2.5, based on bone mineral density (BMD), serves as a diagnostic criterion (Fuggle et al., 2024). Nevertheless, Dual-energy X-ray absorptiometry (DXA) results may not accurately reflect bone strength in individuals with type 2 diabetes. According to the U.S. Diabetes Standard of Care Guidelines 2024, It is recommended that the diagnostic criterion for T-scores in diabetic patients be raised from −2.5 to the equivalent of −2.0 in non-diabetic patients (Brandt et al., 2024). A safety investigation of prevalent anti-osteoporotic medications in patients with type 2 diabetes mellitus and osteoporosis revealed that bisphosphonates (BPs) enhanced bone mineral density and diminished fracture risk irrespective of diabetes status, although did not ameliorate the diabetic condition (Eastell et al., 2020). A cohort analysis indicates that denosumab generates a decreased risk of diabetes (Huang et al., 2024). Given these findings, we are beginning to consider the effects of glucose-lowering drugs on bone metabolism.

Concerning The effect of antidiabetic drugs on bone formation, two medicines have demonstrated osteogenic effects in current trials. Metformin is a primary oral medication for diabetes management that enhances insulin sensitivity in individuals. Recent studies have shown metformin’s beneficial effects on bone metabolism, encompassing molecular biology, morphology, and BMD (Pratley and Gilbert, 2015; Gu et al., 2017; Sheu et al., 2022). This may occur by activating AMP-activated protein kinase (AMPK) and inhibiting the anabolic mTORC1 signalling pathway (Liu B. et al., 2024). The alternative is the glucagon-like peptide-1 receptor agonist (GLP-1RA). GLP-1 is an incretin hormone that promotes insulin secretion in a glucose-dependent way and mediates the gut-bone axis, which is crucial for regulating bone metabolism (Vilaca and Eastell, 2023). In certain animal models and individuals with T2DM and OP, GLP-1RA demonstrated the ability to improve bone turnover markers (Nissen et al., 2019; Skov-Jeppesen et al., 2021), and this may be associated with the Wnt/β-catenin pathway. Which is ultimately regulated by transcription factors such as RUNX2 and facilitates the differentiation of mesenchymal stem cells (MSCs) (Liu H. et al., 2024). In different sources of MSCs, GLP-1 is considered to be an osteoblastic agent (Wang et al., 2023).

BMSCs are capable of proliferation, self-renewal, and multidirectional differentiation (Gao et al., 2022). The direction of differentiation of BMSCs influences the bone-lipid balance during bone repair. Reduced osteoblast activity and increased adipocyte proliferation are key contributors to osteoporosis pathogenesis (Huang et al., 2020). BMSC differentiation is regulated by multiple aspects, including the activation of transcription factors, the transmission of intercellular, and post-transcriptional regulation. The Wnt pathway is a crucial regulatory mechanism for differentiating BMSCs in the present investigation (Wu et al., 2023; Xu et al., 2024). Classical Wnt signaling involves the interaction of lipid-modified Wnt, through a series of biochemical reactions, resulting in elevated nuclear β-catenin levels, ultimately activating Wnt-mediated signaling (Schmiege and Li, 2024). Low-density lipoprotein receptor-associated proteins 5 and 6 (LRP5/LRP6) serve as co-receptors for Wnt ligands. LRP5 and LRP6 may participate in osteogenic differentiation in certain studies (Lim et al., 2021; Wang P. et al., 2024), whereas LRP5 is additionally implicated in cell proliferation and glycolysis (Hong J. et al., 2021). In in vitro studies, GLP-1RA promotes osteogenic differentiation of bone marrow-derived stem cells by increasing β-catenin expression and inducing its translocation into the nucleus and up-regulating the expression of osteogenesis-related genes OCN, RUNX2 or OPN (Meng et al., 2016). In other stem cell classes, such as human or murine-derived adipose-derived stem cells, it has been demonstrated that GLP-1RA upregulates the Wnt signaling pathway and promotes the osteogenic differentiation process and osteogenic gene expression (Lee et al., 2015; Li Y. et al., 2020); it has also been demonstrated that GLP-1RA plays an important role in promoting the proliferation and osteogenic differentiation of periodontal ligament stem cells, and at the same time reduces the periodontal inflammatory response (Pang et al., 2018). In an ovariectomized animal model, exendin-4 treatment of osteoporosis model mice for 4 weeks revealed an increase in bone volume and trabecular parameters in key areas by imaging tests, as well as an increase in bone formation markers (PINP) and suppression of bone resorption marker (CTX) expression (Ma et al., 2013; Pereira et al., 2015). In conclusion, GLP-1RA may inhibit osteoclast activation by promoting the Wnt signaling pathway, activating osteoblast proliferation via the p-AMPK/PGC1α signaling pathway, and inhibiting the OPG/RANKL/RANK signaling pathway via anti-inflammatory, antioxidant, and anti-autophagy pathways [30]. In conclusion, the specific mechanism of the Wnt pathway to promote the osteogenic of BMSCs is still inaccuracy, and the role of semaglutide in BMSCs osteogenesis remains unclear.

We aim to detect the effects and possible mechanisms of semaglutide on the proliferation and osteogenic differentiation of BMSCs. The findings will clarify the regulation of the Wnt/LRP5/β-catenin signaling pathway by semaglutide, thereby establishing an experimental foundation for the effectiveness of GLP-1RA in patients with T2DM and osteoporosis.

## 2 Materials and methods

### 2.1 Cell cultivation, differentiation, and treatment

All experiments received approval from the Ethics Committee of the Third Hospital of Hebei Medical University. Eight specified pathogen-free (SPF) male Sprague-Dawley (SD) rats, aged 4 weeks and averaging 40 g, were procured from the Animal Centre of Hebei Medical University for BMSCs. In summary, adhering to the team’s prior isolation technique for acquiring BMSCs(Wang et al., 2018), the isolated BMSCs were cultivated in a complete medium comprising 1% streptomycin/penicillin, DMEM, and 10% fetal bovine serum (FBS, Gibco, United States). Cells were maintained at 37°C under 5% CO_2_, with medium replenishment every 3 days. Allow cells to reach 75%–80% confluence before passaging. BMSCs were collected after 3 passages for osteogenic induction. BMSCs reached 90% confluence, after which the entire medium was substituted with osteogenic culture (OriCell, Guangzhou, China) and various concentrations (0, 10, 100, or 1,000 nM) of semaglutide were added.

### 2.2 Apoptosis assay

The Annexin V/propidium iodide (PI) apoptosis assay was conducted with the Apoptosis Detection Kit (Lianke Bio, China). Cells were rinsed twice and subsequently digested. 500 μL of 1× Binding Buffer was added to each batch of cells. Single-stained tubes and blank tubes of cells were made concurrently. No stain was introduced to the blank tubes; 5 µL of ANNEXIN V-FITC and 10 µL of PI were administered to the two single-stained tubes, while stains were concurrently added to the sample tubes. Each sample (1 × 10^6^ cells) was analyzed. The percentage of apoptotic was evaluated by analyzing the fluorescent signals of Annexin V-FITC and PI, where early apoptotic cells are characterized as Annexin V-positive and PI-negative, whereas late apoptotic or necrotic cells are identified as Annexin V-positive and PI-positive.

### 2.3 Cell cycle assay

Cell cycle test kits (Lianke Bio, Hangzhou, China) were used, under the manufacturer’s guidelines. After digestion with cells, followed by 1 mL DNA staining solution and 10 µL permeabilising solution, then mixed for 5–10 s. Incubate for 30 min without light; the lowest sample rate is chosen and analyzed using a flow cytometer (ThermoFisher Scientific, United States).

### 2.4 Cell counting Kit-8 (CCK8) assay

Cell viability was measured using the CCK-8 (Beyotime, China). Briefly, BMSCs (3 × 103 cells/well) were incubated in a culture plate. After 6–8 h, a complete medium with DMSO or different concentrations (10, 100, 1000 nM) of semaglutide was added, Cultivate for 3, 5, and 7 days. Mix the appropriate amount of cck-8 solution in the medium, and the cells are placed in an incubator for 1 h. Finally, the absorbance was measured at 450 nm.

### 2.5 Alkaline phosphatase (ALP) staining

To assess ALP expression 7 days after osteogenic induction, an ALP staining kit (Beyotime, China) was used. After 7 days of osteogenic development, BMSCs were rinsed three times with PBS and subsequently kept in 4% paraformaldehyde for 30 min at room temperature. ALP stained and washed twice, and the reaction was halted by the addition of distilled water. Ultimately, the Acquisition of images with an optical microscope (Olympus, Japan), and analyzed using ImageJ to evaluate the level of mineralization.

### 2.6 Alizarin red staining (ARS)

Following 14 days of osteogenic differentiation, cells were fixed with 4% paraformaldehyde for 30 min at room temperature, washed thrice with PBS, and subsequently stained with ARS staining solution (OriCell, Guangzhou, China) for 5–10 min. Upon completion of the staining, cells were rinsed with ddH2O, revealing red spots indicative of calcified nodules. Cetylpyridinium chloride (CPC) was configured to a 10% concentration and added to culture plates after osteogenic differentiation, Measurement of 562 nm absorbance after 1 h.

### 2.7 Oil red O staining

When reaching 75%–80% confluence, BMSCs were supplemented with adipogenic media (OriCell, Guangzhou, China) and grown for 2 weeks. BMSCs were rinsed twice and subsequently fixed for 30 min. Oil Red Stain A and B were mixed and added to the culture plate (Beyotime, China) Staining was conducted for 30 min. The process of lipid droplet generation was seen using microscopy.

### 2.8 Real-time quantitative PCR

Total RNA was isolated from BMSCs cultivated under various conditions employing TRIzol reagent (Ambion, United States), and cDNA was synthesised employing the cDNA HiFiScript Synthesis Kit (Cwbio, China). The cDNA was diluted and employed as a template for future studies. The mRNA levels were measured employing SYBR Green PCR premix (TaKaRa, Japan). GAPDH was used for normalisation, and mRNA expression was quantified using the 2^−ΔΔCT^ technique. The specific information of primer sequences is demonstrated in Table 1.

**TABLE 1:** Primer sequences for RT-PCR.

Gene	Forward sequence (5′→3′)	Reverse sequence (5′→ 3′)
OCN	TGA​GGA​CCA​TCT​TTC​TGC​TCA	TGG​TCT​GAT​AGC​TCG​TCA​CA
Runx2	AGA​TGG​GAC​TGT​GGT​TAC​CG	TAG​CTC​TGT​GGT​AAG​TGG​CC
Wnt3a	GTG​TCA​AGG​CGG​GCA​TCC​AAG	CAG​CGG​AAG​CGA​TGG​CAT​GG
LRP5	ACA​TAG​ACG​GGA​CAA​AGC​GG	CCT​CTC​AAT​ACT​GCG​TCG​CT
β-catenin	CCT​AGC​TGG​TGG​ACT​GCA​GAA	CAC​CAC​TGG​CCG​AAT​GAT​GA
GAPDH	AGT​TCA​ACG​GCA​CAG​TCA​AGG	AGC​ACC​AGC​ATC​ACC​CCA​T

### 2.9 Western blotting

Phenylmethylsulfonyl fluoride (PMSF; Solarbio, China) and RIPA lysate (Beyotime, Shanghai, China) were mixed at 1:100 for lysing cells. After centrifugation, Protein content was quantified with the BCA Protein Assay Kit (Beyotime, Shanghai, China). Electrophoresis transferred Protein samples from 10% SDS-PAGE to polyvinylidene fluoride (PVDF, Sigma, United States). QuickBlockTM Buffer (Beyotime, Shanghai, China) Incubate membrane for 10 min. The samples were subsequently treated overnight at 4°C with primary antibodies: RUNX2, OCN (1:1,000, Proteintech, China), GAPDH (1:3,000, Proteintech, China), Wnt3a, LRP5, and β-catenin (1:1,000, Proteintech, China). Subsequent incubation with secondary antibodies was conducted. Add ECL reagent; an enhanced chemiluminescence system (Kodak, Rochester, United States) was utilized to visualize the signals. The blot’s signal intensity was analysed using ImageJ software (NIH, Bethesda, United States).

### 2.10 Wound healing assay

BMSCs were plated in a culture plate and when 90% confluence was reached. Creation of wounds on monolayer BMSCs, then replaced with complete media containing 1% fetal bovine serum after washing. The extent of wound healing was assessed using ImageJ by capturing microscopic images of the scratches at 0 and 24 h.

### 2.11 Cellular transfection

The cells are cultured until they reach approximately 80% confluence. siRNA targeting LRP5 (GGA​CAG​ACU​CAG​AGA​GAC​CAA) was transferred into the BMSCs with the help of Lipofectamine^®^ 3000 (Invitrogen, United States), and all operations were carried out under the manufacturer’s instructions. After 48 h, cells were lysed for qPCR. Additionally, cells were transfected with an empty vector or a negative control (NC) siRNA, designated as the si-NC group. The siRNA sequences were customised by QianMoBio Co., Ltd., (Shanghai, China).

### 2.12 RNA sequencing and pathway analysis

BMSCs were treated with or without 100 nM Semaglutide in a normal medium for 7 days, and the cells were divided into a control group (A) and an intervention group (B). Total RNA was extracted using Qiagen RNeasy purification columns, following the manufacturer’s instructions and using three samples per group. Ensure purity and integrity of RNA samples by determining concentration with Nanodrop, quantifying and analysing with Qubit 2.0 and Agilent 2100 Bioanalyser. The gene differential expression (DEG) analysis method was used. Sangon Biotech conducted the library construction and sequencing.

|fold change (FC)| values >2 and p values <0.05 were used as criteria for screening DEGs. Venn diagrams are used to elaborate on overlapping genes in different biological processes.

Gene Ontology (GO) and the Kyoto Encyclopedia of Genes and Genomes (KEGG; http://www.genome.ad.jp/kegg) were employed for pathway analysis using enriched R packages. The terms or pathways were mapped and visualized by selecting the top 30 according to the number of genes for which they were enriched.

### 2.13 Immunofluorescence (IF) assay

Following a 14-day culture period, BMSCs were fixed for 20 min. Triton X-100 (Sigma, United States) was diluted to a concentration of 0.1% and used to permeabilise the cell membrane. 1% bovine serum albumin (BSA, ThermoFisher, United States) was used to block non-specific antibody binding. Subsequently, Incubated with primary antibody OCN (1:200, Abcam, United Kingdom) at 4°C overnight. After being washed three times, FITC-labelled rabbit secondary antibodies were incubated at room temperature for 1 h (ThermoFisher, United States) and then fixed with DAPI staining. Finally, Capture images of characteristic stains with a confocal microscope. (Olympus, Japan).

### 2.14 Statistics

Data were replicated in triplicate and expressed as mean ± SD. All graphs and statistical analyses were plotted using GraphPad Prism 10.1.2 software (Santiago, United States). Unpaired t-tests were used between the two groups. A one-way analysis of variance (ANOVA) was conducted to compare more than three groups of data, and a Tukey’s test was employed. Statistical significance was defined as P < 0.05.

## 3 Result

### 3.1 Semaglutide promotes the proliferation of BMSCs

**FIGURE 1:**
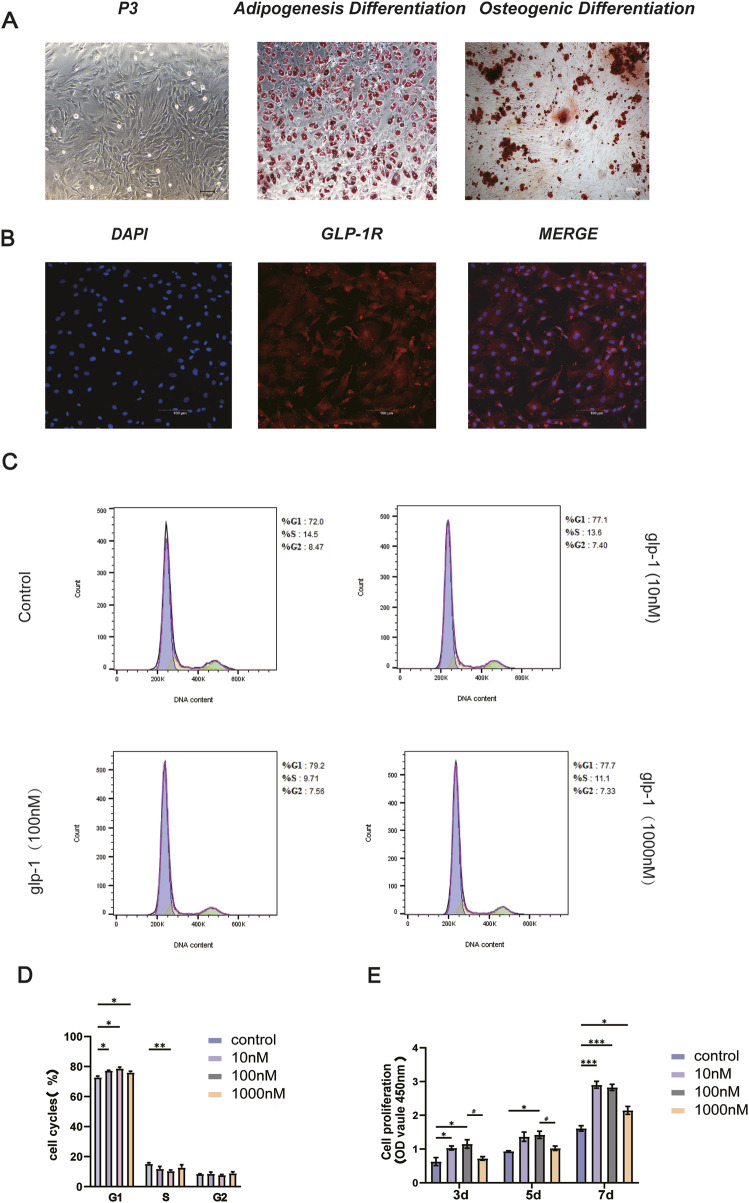
Characterization of BMSCs and GLP-1R expression. Effect of semaglutide on the proliferation of BMSCs. **(A)** Microscopic acquisition of third-generation BMSCs with oil red O and alizarin red staining (Scale bar: 200 μm). **(B)** The presence of GLP-1R on BMSCs is confirmed by immunofluorescence. (Scale bar: 100 μm). **(C, D)** Following a 7-day intervention with varying semaglutide doses, the growth cycle was assessed using a flow cytometry assay **(E)**. For 3, 5, and 7 days, BMSCs were cultivated with varying doses of semaglutide (10, 100, and 1,000 nM). Cell proliferation was then measured using the CCK-8 test kit. SD ± SEM is used to express the data. The data are derived from three independent experiments (n = 3). *p < 0.05, **p < 0.01, and ***p < 0.001 in relation to the control (0 nM). #p < 0.05, compared with the group (100 nM semaglutide).

Bone marrow stem cells (BMSCs) represent a viable approach for osteoporosis treatment because of their self-renewal capacity, pluripotent differentiation, and minimal immunogenicity. Following the extraction and culture of BMSCs, which were then passaged to the P3 generation, the cells displayed morphology characteristic of BMSCs, demonstrating adherent development of elongated spindle-shaped cells with a typical swirling arrangement. Furthermore, osteogenic and adipogenic differentiation was confirmed as positive (Figure 1A), with their surface markers previously validated by our team (Wang et al., 2019). Immunofluorescence detected the GLP-1R (Figure 1B). Previous experiments demonstrated the presence of GLP-1R on BMSCs. Subsequently, to assess the impact of Semaglutide on the proliferation of BMSCs, the toxicity of Semaglutide was evaluated using the CCK-8 assay, and the optimal concentration of Semaglutide was established. Semaglutide at 100 nM significantly enhanced BMSC proliferation (Figure 1E). Flow cytometry assessed the cellular growth cycle at varying dosage concentrations (10, 100, and 1,000 nM). The findings indicated that the G1 and S phases of the cell cycle were markedly elevated (p < 0.05) following the administration of 100 nM Semaglutide, confirming the results of the CCK-8 experiment (Figures 1C, D).

### 3.2 Semaglutide promotes osteogenic differentiation of BMCSs

**FIGURE 2:**
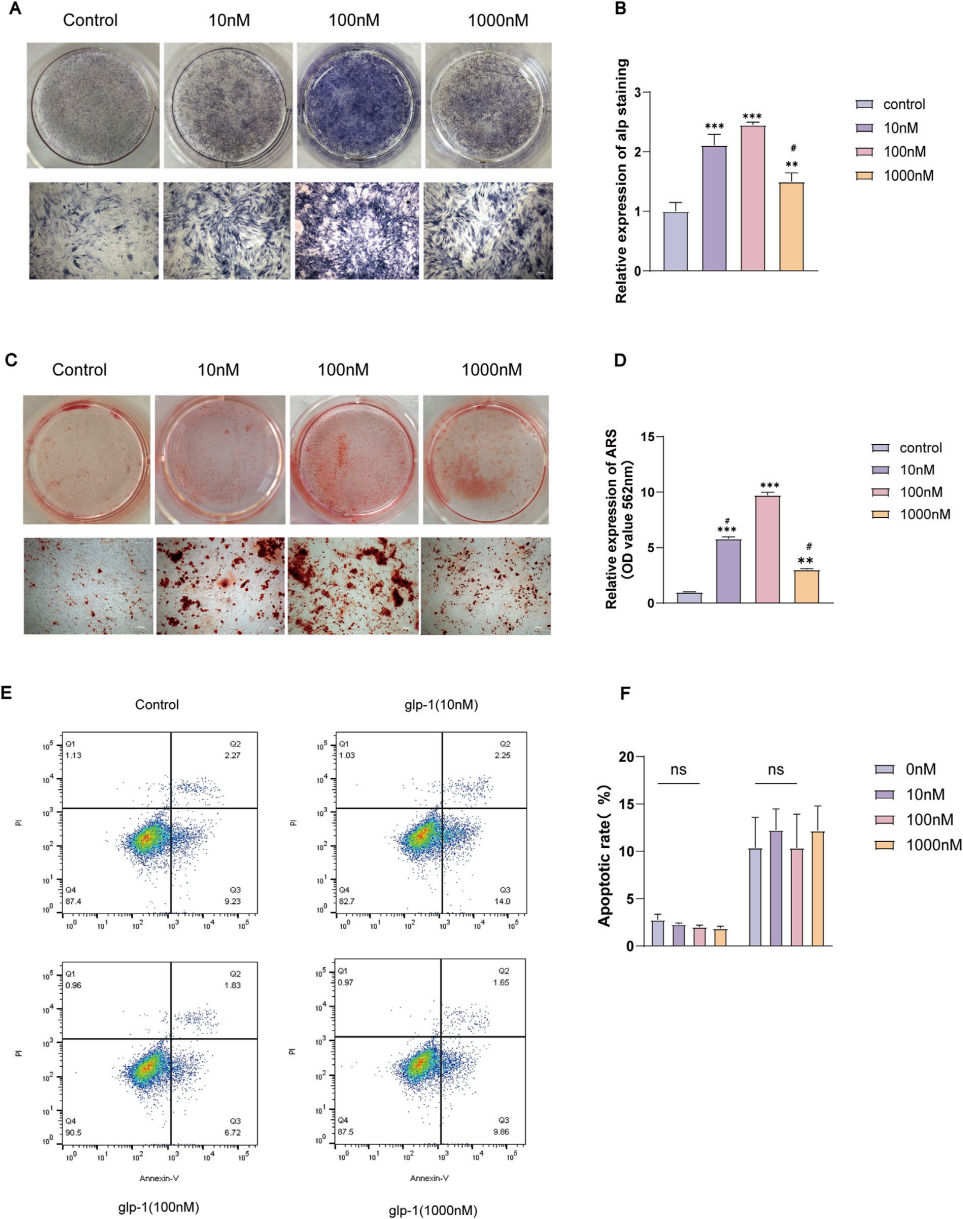
Semaglutide promoted osteogenic differentiation of BMSCs and had no significant effect on apoptosis. **(A, B)** Treatment with varying concentrations of semaglutide for 7 days, followed by ALP staining and quantification, was used to evaluate osteogenic differentiation. Mineralization levels were measured using Alizarin Red S staining **(C, D)** following a 14-day Semaglutide intervention. **(E, F)** Following 7 days of semaglutide, the apoptosis kit was employed to quantify the levels of apoptosis in the BMSCs. In comparison to the control (0 nM), The data are derived from three independent experiments (n = 3). *p < 0.05, **p < 0.01, and ***p < 0.001; ns (not significant). #p < 0.05 compared with the group (100 nM semaglutide). (Scale bar: 200 μm).

To examine the effect of GLP-1RA on the osteogenic differentiation of BMSCs, different doses of semaglutide were administered to the cells for durations of 7 and 14 days. ALP staining for 7 days indicated a significant increase in ALP activity at 10 and 100 nM (Figures 2A, B), and ARS staining for 14 days indicated that mineralisation was most pronounced at 100 Nm (Figures 2C, D). Nonetheless, both demonstrated the unanticipated reduction in ALP activity and calcium salt deposition at 1,000 nM (Figures 2A–D). To enhance the drug’s cellular safety assessment, the CCK-8 assay was supplemented with an apoptosis assay, which indicated no significant variation in apoptosis across different concentrations (Figures 2E, F). The optimal concentration for enhancing proliferation and osteogenic differentiation was identified as 100 nM, in agreement with earlier research (Li Y. et al., 2020). Consequently, the doses employed in the subsequent examinations were uniformly 100 nM.

### 3.3 Semaglutide promotes BMSCs migration and increases osteogenesis-related gene expression

**FIGURE 3:**
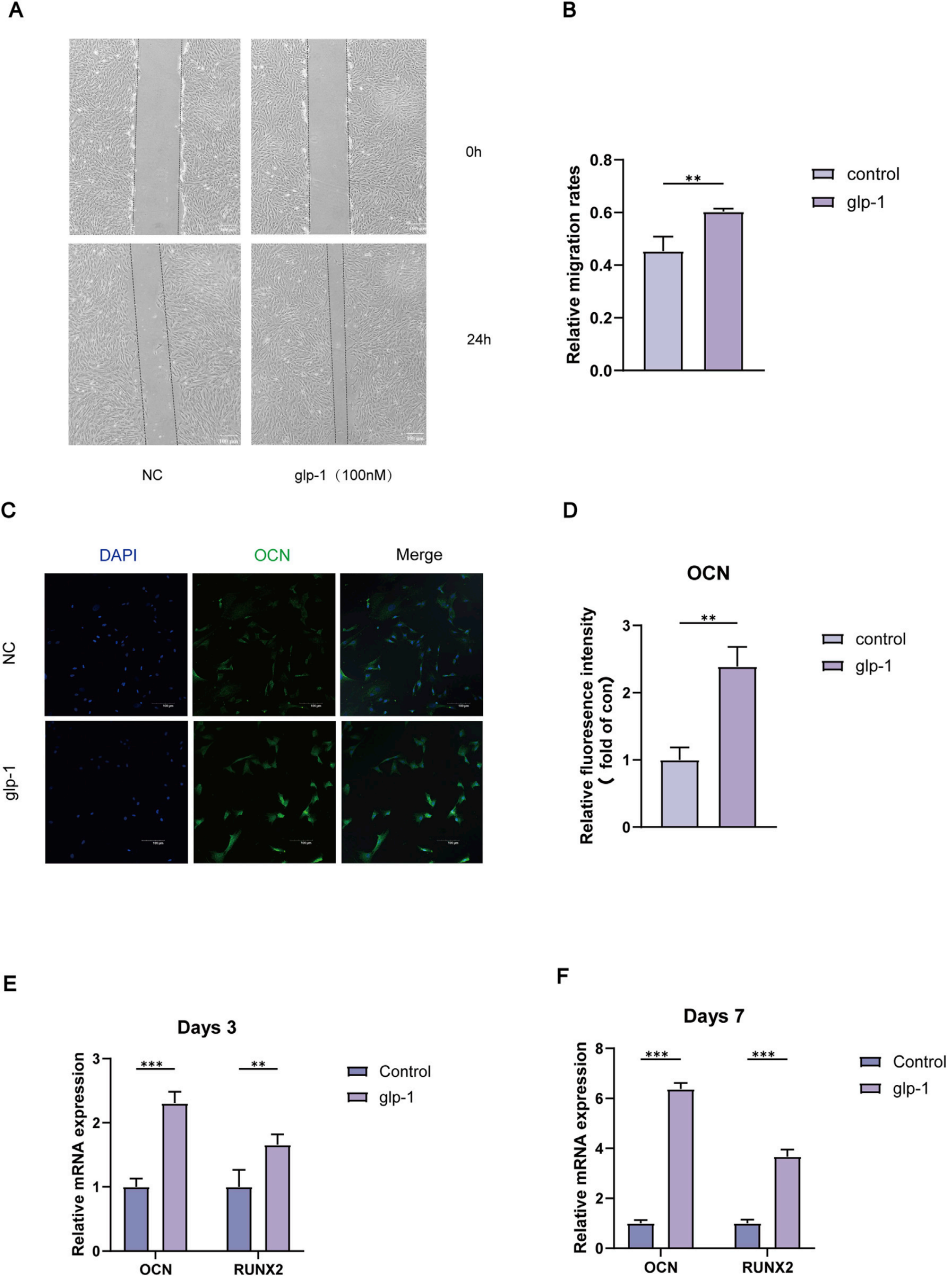
Semaglutide promotes BMSCs migration and osteogenic genes of OCN and RUNX2 expression. **(A, B)** The migration rate of BMSCs after 0 and 24 h is determined using the wound healing test. **(C, D)** Immunofluorescence for semi-quantitative analysis of OCN and microscopic representative images of DAPI (blue) and OCN (green) were captured. (Scale bar: 200 μm). **(E, F)** After three and 7 days, OCN and RUNX2 mRNA expression was detected using real-time RT-PCR. *p < 0.05, **p < 0.01, and ***p < 0.001 with the control (0 nM).

To clarify the impact of GLP-1RA on BMSCs, we examined the migratory potential of BMSCs following semaglutide administration. A scratch wound healing assay was conducted at 0 h and 24 h (Figure 3A). The findings indicated that 100 nM of semaglutide significantly enhanced cell migration relative to the control group (p < 0.05) (Figure 3B). We investigated the effect of semaglutide on bone differentiation markers, observing that mRNA levels of OCN and RUNX2 were markedly elevated in the treatment group at 3 and 7 days. Moreover, the expression levels demonstrated a progressive increase with the extension of the intervention duration (Figures 3E, F). Additionally, the relative expression of OCN in IF was determined, which exhibited a consistency with the trend observed in mRNA expression (Figures 3C, D).

### 3.4 Bioinformatics analysis of DEGs in BMSCs

**FIGURE 4:**
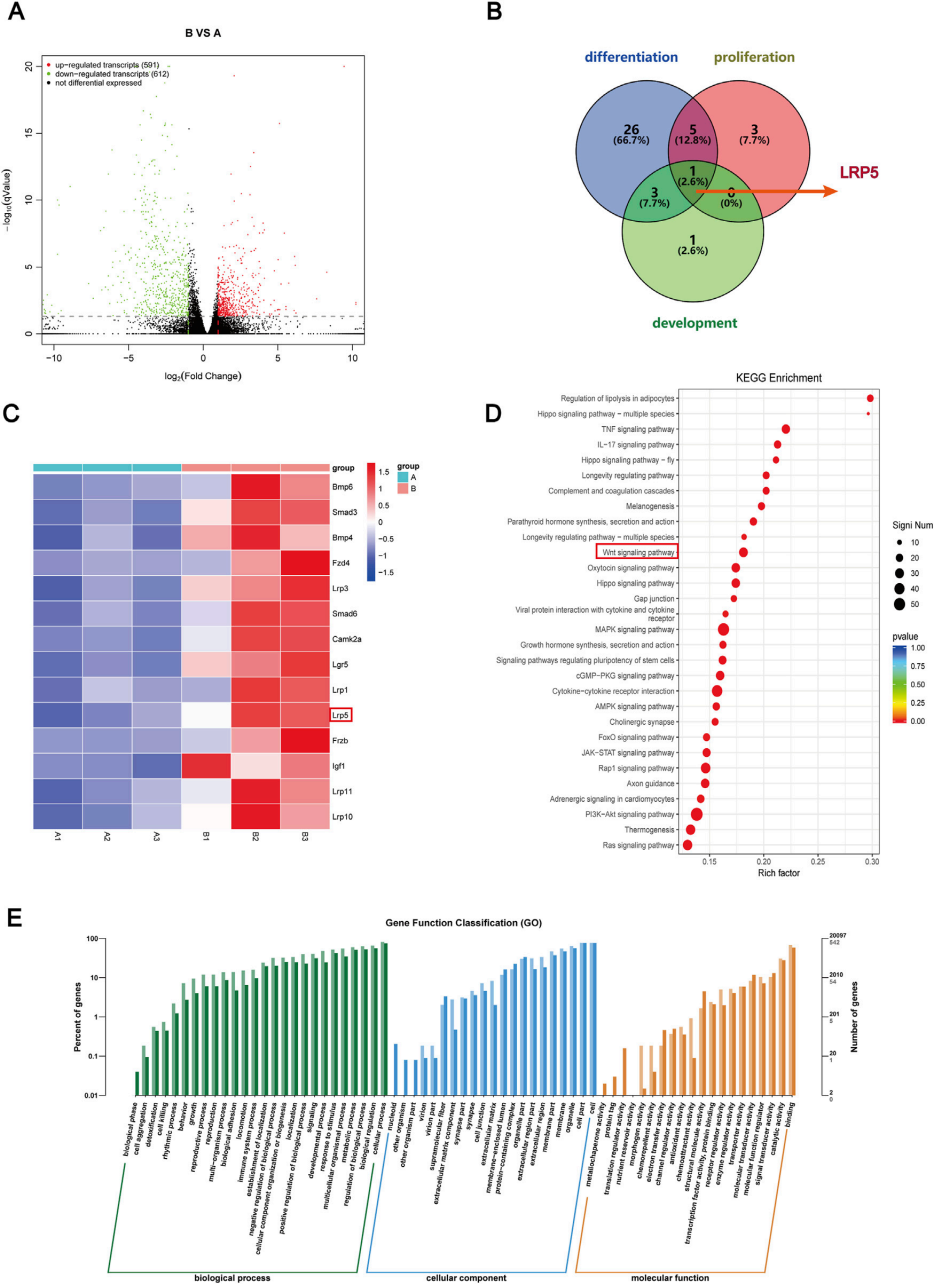
Bioinformatics analysis after semaglutide treated with BMSCs and Screening the candidate DEGs. **(A)** Screening for DEGs after semaglutide treatment of BMSCs at 7 days, compared with the blank group. Represented by a Volcano plot. The screening criteria are |FC| value > 2, and p-value < 0.05. Red and green dots denote upregulated and downregulated genes, respectively **(B)** A Venn diagram was drawn of DEGs enriched for three biological processes, osteoblast differentiation, osteoblast development, and osteoblast proliferation, and screened for overlapping DEGs (LRP5). **(C)** Heap mapping of DEGs in each sample. Lines indicate genes and columns indicate samples (**(A)** represents the control group and **(B)** represents the semaglutide intervention group). Red and blue colours indicate up- and downregulation, respectively. **(D)** The top 30 analyses’ KEGG pathway enrichment. The number of genes is indicated by the circle’s size. **(E)** Top rankings in GO enrichment analyses are plotted, and the vertical coordinates indicate the number and percentage of genes enriched.

To explore the possible mechanism by which GLP-1RA influences the osteogenic differentiation of BMSCs, the DEGs of BMSCs from the semaglutide-treated (B) and control (A) groups were analyzed using RNA sequencing. The screening criteria of |FC| value >2 and p < 0.05 resulted in 591 upregulated and 612 downregulated DEGs in post-treatment (B group), as illustrated by the volcano plot (Figure 4A). Each sample is from an individual rat. The GO analysis showed that the identified DEGs were mainly enriched in processes that are associated with the growth and development of cells and tissues, as well as immune and intercellular signaling pathways (Figure 4E). Thus, it can be inferred that semaglutide may regulate the proliferation of BMSCs. KEGG pathway analysis revealed involvement in several pathways, including the Hippo, Wnt, MARK, and stem cell regulation-related signaling pathways, along with the regulation of hormonal pathways such as parathyroid hormone and growth hormone (Figure 4D).

### 3.5 Screening and validation of candidate DEGs and docking with drug molecules test

The top 30 in GO and KEGG analyses provided some evidence for osteogenic differentiation and osteoblast proliferation. To further screen overlapped DEGs for osteogenic proliferation and differentiation under semaglutide treatment, we used osteoblast proliferation, osteoblast differentiation and osteoblast development as the main biological processes to screen overlapped DEGs as indicated by the Venn diagram (Figure 4B) and obtained the key gene LRP5. LRP5 primarily encodes the transmembrane LDL receptor, which facilitates Wnt protein signaling and participates in other processes, including skeletal development. The LRP gene family is significantly upregulated in KEGG when further enriched for the Wnt pathway, including the LRP5 gene (Figure 4C). Following the identification of the potential involvement of GLP-1 in the Wnt/LRP5 signalling pathway, the interaction between GLP-1 and LRP5 was predicted by employing computer software. The results indicated a high degree of affinity and binding between GLP-1 and LRP5, thereby providing a theoretical foundation for the subsequent experimental procedures (Supplementary Figure S1).

### 3.6 The inhibitory effect on BMSCs osteogenic differentiation following the knockdown of LRP5 was alleviated by semaglutide

**FIGURE 5:**
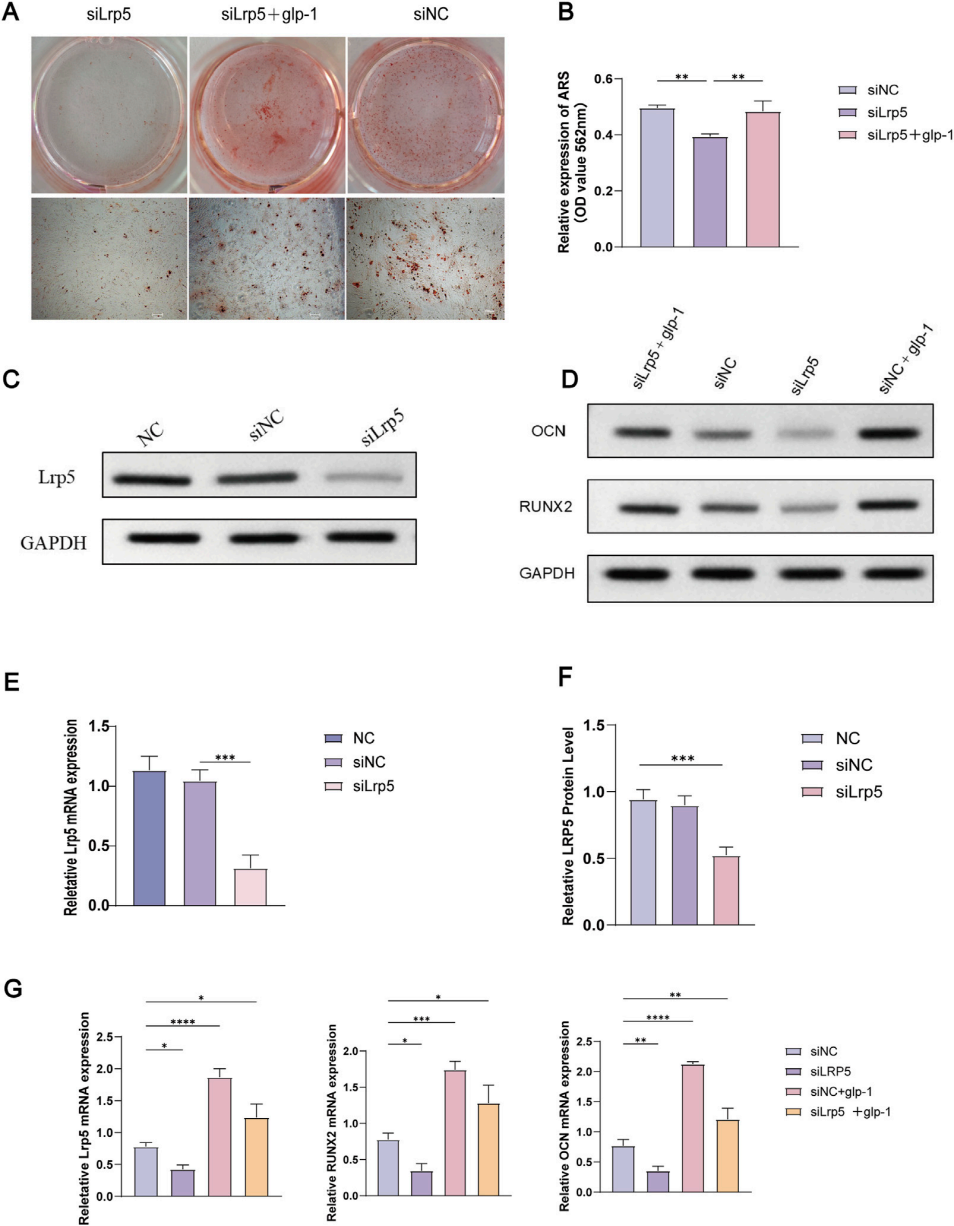
Knockdown of the LRR5 gene inhibits osteogenic differentiation, and semaglutide partially reverses this effect. **(A, B)** Following the knockdown of LRP5, BMSCs underwent osteogenesis for 14 days with or without semaglutide, were stained with alizarin red S, and quantified using 10% CPC. **(C)** Western blotting was employed to detect LRP5 protein expression following 72 h of siRNA intervention. **(D)** The expression of the OCN and RUNX2 proteins was analysed by Western blotting in 4 groups (siNC, LRP5, siNC+GLP-1, LRP5+GLP-1), which were co-incubated for 72 h with or without Semaglutide. **(E–G)** The relative expression of LRP5, OCN and RUNX2 mRNA and protein was detected by RT-PCR and Western blotting following knockdown of LRP5 at 48 h later (n = 3). *p < 0.05, **p < 0.01, ***p < 0.001, compared with the control group (siNC).

By the manufacturer’s protocols, LRP5 mRNA levels were diminished by 70% as determined 48 h following siRNA intervention (Figure 5E). Similarly, the LRP5 protein level was significantly reduced following the knockdown of LRP5 (Figures 5C, F, Supplementary Table S2). Additionally, a notable decline in calcium salt mineralisation was evident in the si-LRP5 group (Figure 5A). Calcium salts were extracted using 10% CPC were measured at 562 nm. The results showed that the siRNA group showed a significant decrease in OD value, which was statistically different from the blank group (P < 0.05). However, the OD values were increased after 14 days of semaglutide treatment. This resulted in elevated and statistically different (p < 0.05) OD values in the si-LRP5 + GLP-1 group compared with those in the si-LRP5 group (Figure 5B, Supplementary Table S3). The role of semaglutide in BMSCs in the NC group was also investigated. As anticipated, Semaglutide treatment significantly enhanced osteogenic potency compared to the si-NC and NC groups (Figures 5D, G).

### 3.7 Semaglutide activates the Wnt/LRP5/β-catenin signaling pathway and promotes osteogenic differentiation

**FIGURE 6:**
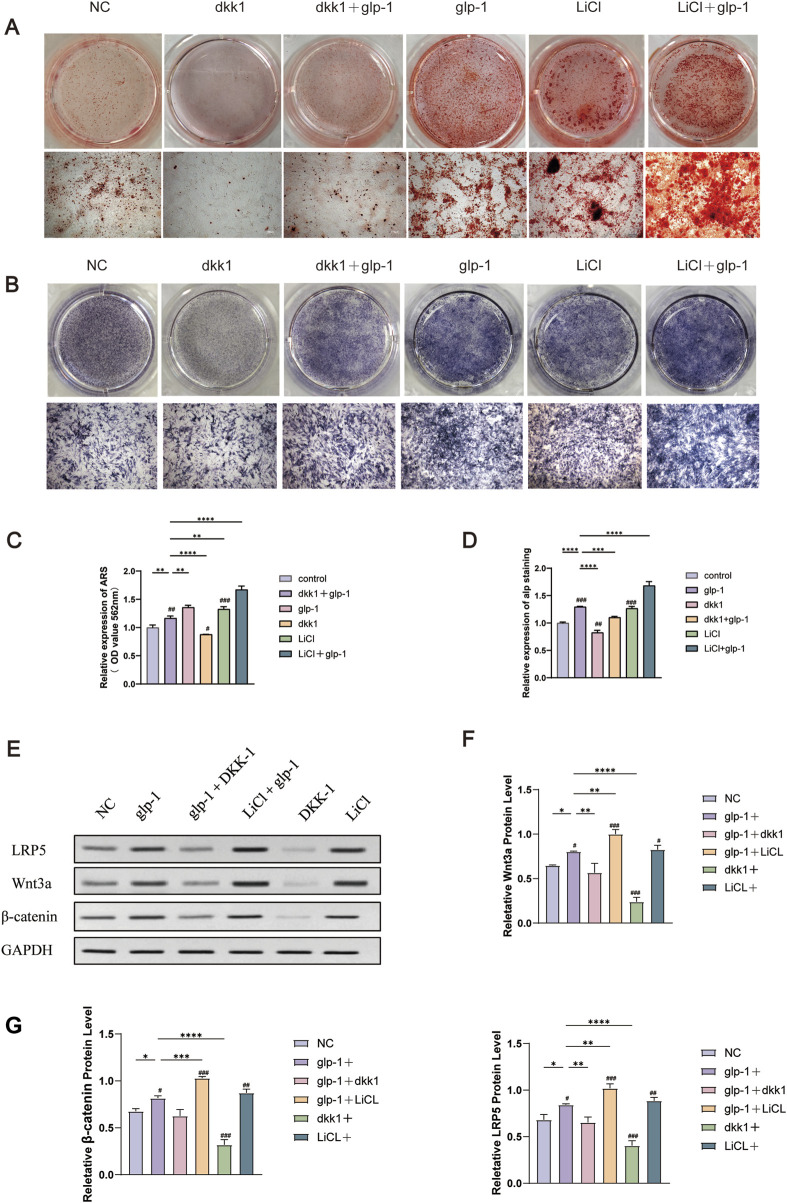
Semaglutide activates the Wnt signaling pathway and partially reverses the inhibitory effect of dkk1 on osteogenesis. **(A)** Alizarin red staining and the relative level of mineralization for 14 days were measured in six groups (Control, glp-1, glp-1+dkk1, dkk1, LiCl and LiCl+glp-1) (n = 3). **(B)** Six groups of ALP staining at 7 days. **(C, D)** Semi-quantitative statistical analyses after staining with alizarin red and ALP. **(E–G)** The relative protein expression of the WNT pathway (Wnt3a, β-catenin, and LRP5) in four groups (n = 3). *p < 0.05, **p < 0.01, ***p < 0.001. Compared with the group (100 nM semaglutide). #p < 0.05, #p < 0.01, ###p < 0.001 compared with the control group.

**FIGURE 7:**
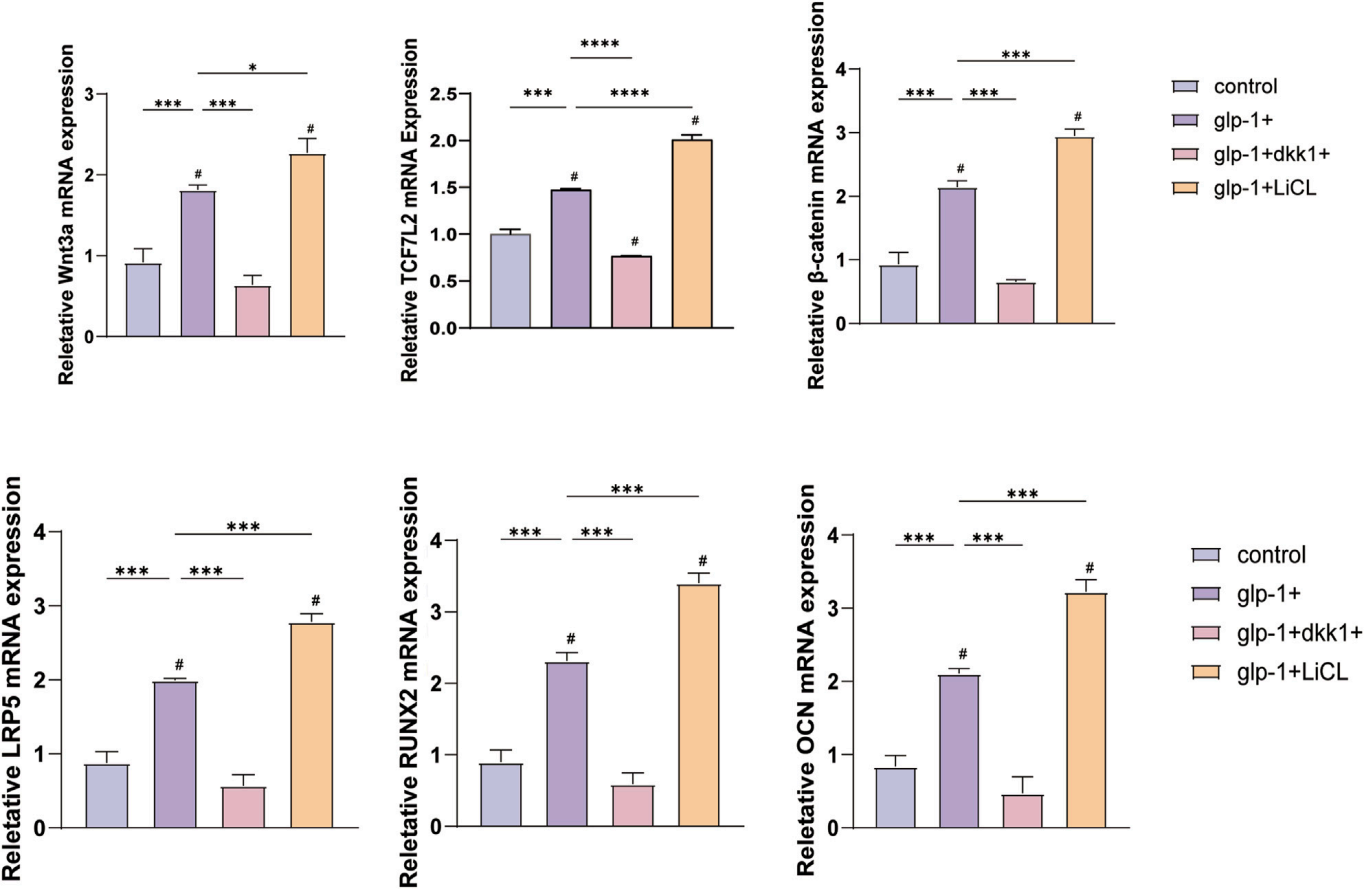
DKK1 and LiCI were observed to inhibit or promote the expression of Wnt3a, β-catenin, LRP5, OCN, TCF7L2 and RUNX2 mRNA. The addition of semaglutide was found to enhance the effect of LiCI and suppress the effect of DKK1. *p < 0.05, **p < 0.01, ***p < 0.001. Compared with the group (100 nM semaglutide). #p < 0.05, #p < 0.01, ###p < 0.001 compared with the control group.

The subsequent experiment aims to determine whether semaglutide promotes osteogenic differentiation of BMSCs through the Wnt/LRP5/β-catenin signaling pathway. DKK1 and LiCl were selected for addition to the medium to elucidate the impact of semaglutide on BMSCs further. DKK1 and LiCl at 200 ng/mL and 20 nM (Lu et al., 2016; Xu et al., 2022). ALP staining and ARS staining after DKK treatment showed a significant decrease in both ALP activity and calcium salt deposition compared to controls. This was reversed by semaglutide intervention and restoration of levels to control group levels. Furthermore, the calcium deposition of BMSCs was found to be significantly increased after LiCl intervention, and the effect of matrix mineralization was further enhanced by the co-intervention of GLP-1 and LiCl. A comparable outcome was observed in the ALP staining results. Both glp-1 and LiCl were found to promote osteogenesis in BMSCs, and their combined administration resulted in a cumulative effect. (Figures 6A–D). In terms of the Wnt/LRP5/β-catenin signaling pathway, DKK1 was able to repress the expression of LRP5, β-catenin, and transcription factor 7-like 2 (TCF7L2) on mRNA, whereas the addition of LiCl was able to promote their expression. The negative effect of DKK1 was partially reversed by semaglutide, whereas lithium chloride significantly upregulated the Wnt pathway (p < 0.01) in all other groups; DKK-1 and LiCl could promote and inhibit the expression of related Wnt3a, LRP5 and β-catenin at the protein level (Figures 6E–G; Figure 7).

## 4 Discussion

Extensive studies have shown that diabetes has long-term effects on the skeleton, Increased risk of bone disease. Among these, the effects of T1DM on bone microarchitecture are widely recognized, whereas in T2DM despite normal bone mass, fracture risk and increased bone fragility remain major challenges (Wongdee and Charoenphandhu, 2015; Hofbauer et al., 2022). MSC transplantation shows potential in regenerative medicine and diabetes treatment as exhibits multifunctionality, self-renewal capabilities, and multidirectional differentiation (Thakkar et al., 2015).

The relationship between the gut and the skeleton has been extensively studied, highlighting its significance within the overall physiology of the organism. Semaglutide is a widely utilized long-acting GLP-1RA, primarily indicated for the treatment of T2DM. GLP-1RA have been shown to affect various tissue organs, including the cardiovascular and urinary systems (Chen K. et al., 2024), Some studies have confirmed that GLP-1R is present in osteoblasts and their progenitors (Pacheco-Pantoja et al., 2011). Recent research findings that GLP-1 may positively affect bone metabolism (Hansen et al., 2017; Ren et al., 2024). Cheng et al. reported that GLP-1 exhibited an osteogenic effect in diabetic rats, leading to enhanced bone strength and increased mRNA expression of bone-forming markers (Cheng et al., 2022). Furthermore, *In vitro* studies indicate that GLP-1 has osteogenic effects on various cellular sources, such as osteoblasts and bone precursor cells, consistent with our findings (Deng et al., 2019; Habib et al., 2022). Currently, there is no clinical evidence supporting the hypothesis that GLP-1 and its analogues contribute to bone formation in diabetic patients.

GLP-1RA has been shown to promote osteogenic differentiation and proliferation *in vitro*, this is consistent with our findings (Patel and Patel, 2017). Our results demonstrate that semaglutide can affect osteogenic differentiation and promote the proliferation of BMSCs. Specifically, we selected concentrations of semaglutide spanning from 10 to 1,000 nM for our CCK-8 proliferation assay, which was based on our previous experiments (Li Z. et al., 2020). The results demonstrated that 10, 100, and 1,000 nM of semaglutide could promote the proliferation of BMSCs; however, 100 nM was the most effective concentration. Furthermore, the cell growth cycle was determined by flow cytometry, which revealed that 100 nM of semaglutide could significantly increase the proportion of cells in the G1 and S phases. This suggests that the effect of semaglutide on BMSCs may be reflected in the pre-cell division phase. Consequently, 100 nM was identified as the optimal dose.

The effects of semaglutide were also reflected in the progress of osteogenic differentiation and the regulators of osteogenic differentiation (RUNX2, ALP, OCN). ALP is an early marker of osteogenesis and promotes matrix mineralisation during osteogenesis (Sharma et al., 2013). As a key bone transcription factor, RUNX2 plays an important role in the initiation of osteogenic differentiation and bone formation (Park et al., 2019). In conclusion, the ALP and ARS staining results demonstrated that 100 nM of semaglutide stimulates BMSCs osteogenic differentiation. To identify any adverse effects of semaglutide on BMSCs, apoptosis was determined by flow cytometry. This demonstrated that 100 nM semaglutide was not potentially cytotoxic. It can therefore be concluded that semaglutide not only increased ALP activity, thereby increasing the number of calcium salt deposits, but also promoted the expression of OCN and RUNX2 at the mRNA and protein levels. This finding is consistent with those of previous reports (Meng et al., 2016), In addition, liraglutide was found to enhance osteogenesis in BMSCs through the BMP2/Smad/Runx2 signalling pathway and increased new bone formation after bone grafting, independent of blood glucose levels (Wang Y. et al., 2024). In a de-ovulated rat model experiment, continuous subcutaneous injection of semaglutide improved bone microarchitecture and upregulated the Wnt signalling pathway (Abo-Elenin et al., 2024). When considered alongside the results of our own earlier study (Gao et al., 2018), provides compelling evidence that semaglutide can promote the osteogenic differentiation process in BMSCs.

RNA sequencing conducted after treatment with semaglutide revealed DEGs. The key gene LRP5 was identified through bioinformatics GO analysis of overlapping genes, in three major biological processes. Next, KEGG enrichment analysis revealed the potential signaling pathway, specifically the Wnt pathway. To conduct a thorough investigation into the mechanism of LRP5 alteration after GLP-1 intervention, we employed AutodockVina software to undertake a comprehensive analysis of the molecular docking experiments of GLP-1 on LRP5 protein. Our findings revealed that GLP-1 established hydrogen bonds with the LRP5 proteins at positions ASN-416, VAL-709, THR-672, GLU-714, ARG-977, GLU-393, and TYR-1511. The resultant overall affinity score between the two proteins was determined to be −12.1 kcal/mol, signifying a robust binding force between them. This provides a theoretical foundation for subsequent mechanistic studies of the role of GLP-1 on Wnt/LRP5/β-Catenin at the molecular level.

LRP5 is vital to the Wnt/beta-catenin signaling pathway, which is involved in a variety of biological processes. And regulates bone metabolism (Kobayashi et al., 2024). A preliminary investigation indicated that the activation of the Wnt pathway significantly influenced bone metabolism in mice through various experimental models (Baron and Kneissel, 2013). Wnt ligand proteins cooperate with LRP5/LRP6 as co-receptors and a seven-transmembrane-spanning protein known as Frizzled, resulting in a complex that triggers various ubiquitination and phosphorylation effects. The dissociation of the disrupting complex prevents the phosphorylated degradation of β-catenin, thereby promoting the expression of Wnt target genes (Arya et al., 2024).

The Wnt/β-catenin signaling pathway is critical for bone homeostasis. Mutating more than one allele of LRP5 is associated with serious consequences, while heterozygous loss-of-function variations may result in milder manifestations of the condition (Littman et al., 2023). The activation of the Wnt signaling pathway has been shown to positively impact many processes associated with BMSCs proliferation, migration, and differentiation (Liu F. et al., 2023). Certain herbal anti-osteoporosis therapy strategies may also be associated with the Wnt pathway (Gao et al., 2021; Liu J. et al., 2023). Although some research has shown that GLP-1RA can facilitate osteogenesis, the precise mechanism of action is still debated. The study aims to ascertain the involvement of the Wnt/LRP5/β-catenin pathway in the proliferation and differentiation of BMSCs. It was previously shown that 100 nM semaglutide promotes calcium salt deposition and mRNA expression of the osteogenic factors OCN and RUNX2. On this basis, we elected to silence the LRP5 gene with siRNA. After knocking down LRP5, the promoted bone formation effect of semaglutide was suppressed, both in terms of calcium salt deposition and the expression of the osteogenic markers OCN and RUNX2. Subsequently, it was observed that 100 nM semaglutide was able to partially reverse the negative effect of knocking down LRP5, Therefore, we suggest that LRP5 is involved in the osteogenesis process promoted by GLP-1. In this study, we also included DKK1, an inhibitor of the Wnt/β-catenin signalling pathway, and LiCl, a pathway enhancer, to ascertain whether this osteogenic effect could be blocked or enhanced. DKK1 inhibited the active expression of ALP and the accumulation of mineralised nodules and additionally affected the levels of mRNA and protein expression of genes that are markers of osteogenesis. Conversely, LiCl further enhanced the osteogenic effect. Subsequently, we observed the genes of focus in this experiment, which were Wnt3a, LRP5 and β-catenin. The expression of mRNA and protein levels were suppressed under the treatment with DKK1, and this effect could be reversed by semaglutide; whereas, the addition of LiCl resulted in the further upregulation of Wnt3a, LRP5, and β-catenin mRNA and protein levels. This is due to certain experimental findings (Abo-Elenin et al., 2024; Chen Z. et al., 2024). In our previous experiments, we demonstrated that liraglutide was able to promote osteogenic proliferation and differentiation through the Wnt pathway network crosstalk. Additionally (Hou et al., 2020), Additionally, it has been suggested that other pathways may also be involved in osteogenic calcification. We investigated Wnt/LRP5/β-catenin as an upstream signaling pathway for the first time and further identified the key role of LRP5 in Wnt, which can be reflected in some of the current studies (Esen et al., 2013; Cai et al., 2020; Yu et al., 2022b). In addition, it has been established that the activation of the WNT signalling pathway results in an increase in β-catenin levels and the promotion of the binding of nuclear β-catenin to TCF/LEF. This ultimately leads to the upregulation of the expression of osteogenesis-related target genes. The protein encoded by the transcription factor 7like 2 (TCF7L2) gene is TCF4, which plays an important role in the Wnt signalling pathway and promotes the synthesis and secretion of glucagon-like peptide-1 (GLP-1), which in turn lowers blood glucose levels. Another *in vivo* study found that *in vivo* knockdown of the TCF7L2 gene in mouse adipocytes resulted in impaired glucose tolerance and reduced GLP-1 levels (Nguyen-Tu et al., 2020). *In vitro* experiments showed that high glucose toxicity inhibited GLP-1 secretion by enteroendothelial cells and suppressed β-catenin and TCF7L2 levels, suggesting an important role for TCF4 (TCF7L2) in glucose homeostasis and type 2 diabetes development (Hong J.-H. et al., 2021). We found that the activation of Wnt/β-Catenin/TCF7L2 also plays an important role in osteogenic differentiation and improvement of bone metabolism. In some studies, the activation of the Wnt signalling pathway and the increased expression of TCF7L2 have been shown to promote osteogenic effects. However, there are fewer studies on the effects of GLP-1 on the Wnt/LRP5/β-catenin signalling pathway and TCF7L2 gene association, and further studies are still needed to clarify the relationship between the two. Our conclusions show that GLP-1RA might alter the expression level of TCF7L2 in the nucleus. Combined with the current studies, our findings may prove to be a transit point for bridging GLP-1 and TCF7L2-related studies (Karaman et al., 2020; Shen et al., 2020; Zhang et al., 2023). In addition, LRP5 is another object that we focused on in our experiments. The importance of LRP5 may not only be reflected in the process of osteogenesis but also be related to some tissue physiological states and pathological diseases (Ma et al., 2023; Wang F. et al., 2024).

The roles of LRP5 and LRP6 in osteogenesis remain debated. A recent study suggested that the inhibition of osteogenesis caused by LRP5 deficiency could be restored by supplementation of recombinant Wnt3a protein. However, lipid deficiency inhibited the osteogenic differentiation of BMSCs. LRP5 plays a key role in the lipid uptake of BMSCs and promotes osteogenesis (Lin et al., 2021). Moreover, the SP7 (osterix) binding site was found on the LRP5 promoter, indicating that LRP5 plays a regulatory role in early osteogenesis (Yu et al., 2022a). For the Wnt pathway, LRP6 may also be a critical factor. It has also been reported that Wnt/LRP6/β-catenin is involved in bone trabeculation in osteoblast cell lines (Xiong et al., 2024). In animal models, the co-deletion of LRP5 and LRP6 resulted in severe local and global bone loss in mice (Lim et al., 2021). In a recent study, increased LRP5 expression promoted the osteogenic effects of the Wnt/β-catenin signalling pathway, with consequent effects on bone (Chen et al., 2022). In conclusion, the present study demonstrated that semaglutide promoted the proliferation and osteogenic differentiation of BMSCs through the Wnt/LRP5/β-catenin signaling pathway, and proved that LRP5 may be a key factor in GLP-1 promoting osteogenesis, which has certain positive implications for improving BMSCs function in OP patients with T2DM.

## 5 Conclusion

**FIGURE 8:**
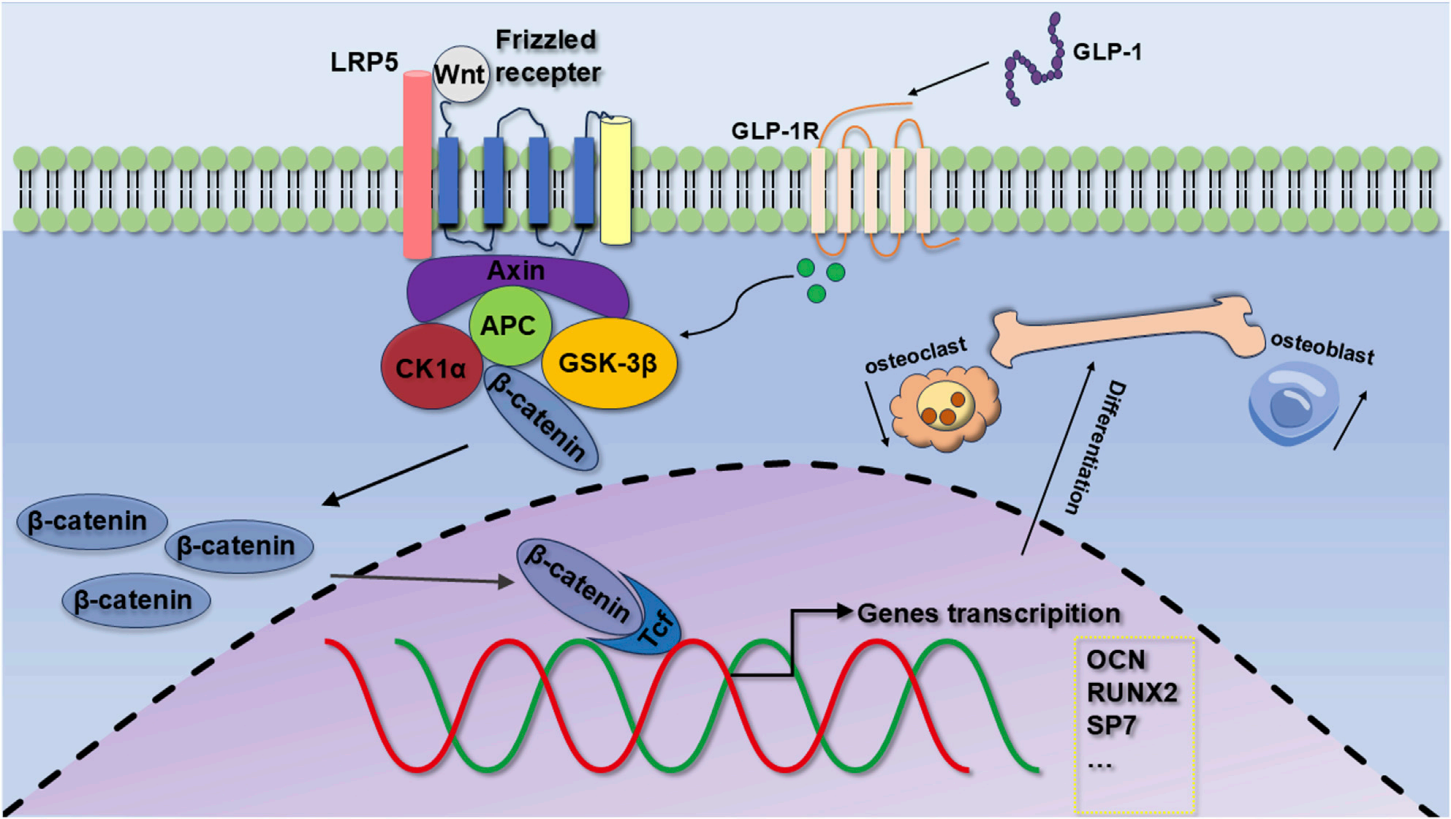
Mechanisms of osteogenic differentiation of GLP-1RA on BMSCs. GLP-1 promotes the proliferation of BMSCs, the expression of OCN and RUNX2, and osteogenic differentiation by regulating the Wnt/LRP5/β-catenin signaling pathways.

Our results suggest that semaglutide significantly increases the proliferation of BMSCs through the modulation of the cell cycle; Semaglutide upregulates the expression of key osteogenic markers (OCN, RUNX2) and increases alkaline phosphatase (ALP) activity and calcium salt deposition levels; Semaglutide promotes bone formation through the activation of the Wnt/LRP5/β-catenin signaling pathway (Figure 8). RNA sequencing and functional validation experiments demonstrated that LRP5 serves as a critical target within this pathway. Semaglutide was found to partially counteract the inhibitory effects of DKK1, a Wnt inhibitor, on osteogenic differentiation, while synergistically enhancing osteogenic activity in conjunction with LiCl, a Wnt agonist. The findings indicate that semaglutide may influence the activity of BMSCs and could be an appropriate medication for people with type 2 diabetes and osteoporosis. Furthermore, it provides considerable promise in the prevention and treatment of T2MD combined with OP, as well as *in vivo* transplantation to enhance osteogenesis.

## 6 Limitations

Nevertheless, the study has limitations. First of all, osteoporosis is a disease affected by many factors. This *in vitro* study necessitates validation through *in vivo* models, and the scope of conclusions is limited. We used to further validate our results by analysing existing experiments regarding the role of GLP-1RA in animal models. In a review of GLP-1RAs in the rat model of osteoporosis, liraglutide could partially improve bone tissue microstructure and bone-related parameters on imaging. It could increase OCN and PINP and decrease serum CTX-1. Liraglutide could also activate osteoblast proliferation by promoting the Wnt/LRP5/β-catenin and p-AMPK/PGC1α signalling pathways and inhibit osteoclast activation by inhibiting the OPG/RANKL/RANK signalling pathway, thereby inhibiting bone resorption (Wu et al., 2024). This is strong evidence that GLP-1 promotes osteogenesis through the Wnt/LRP5/β-catenin signalling pathway. As a new GLP-RA drug, there are few studies on semaglutide. In a recent study of semaglutide in ovariectomized rats, the key role of Wnt/β-Catenin signaling was again reflected. The results showed that compared with the model group, the medium (150 mcg/kg) and high (300 mcg/kg) dose groups of semaglutide significantly increased GLP-1R expression, BMD and serum levels of bone formation markers (BALP, OCN). It is noteworthy that in validating the canonical Wnt pathway, it was found that at medium and high doses, the expression of GLP-1R was significantly increased. BMP-2 and β-Catenin were significantly increased, and the increase was more significant at high doses (Abo-Elenin et al., 2024). The present *in vivo* study explains that semaglutide may improve bone metabolism through regulation of the Wnt/β-catenin signalling pathway, which is consistent with our previous results. At the same time, we also understand that there is still controversy about the effect of semaglutide on bone metabolism, and animal studies are a reliable way to improve credibility. Secondly, although the mechanism proposed in this study was validated in BMSCs, the *in vivo* environment is complex and affected by multiple factors, involving multiple pathways and metabolism, making it difficult to conclude that the GLP-1-activated Wnt pathway plays a decisive role in osteogenesis. Finally, the translational relevance of the 100 nM dose remains uncertain due to discrepancies with clinical pharmacokinetics, and the clinical safety at this dose under long-term use still needs to be further evaluated. Follow-up studies should focus on (1) systematic validation of the osteoprotective effects of semaglutide in combination with *in vitro* and *ex vivo* models, (2) collaborative multi-omics analyses to reveal its regulatory network, (3) preclinical and clinical trials to assess its ameliorative effects on bone mineral density and fracture risk in patients with diabetic osteoporosis. We hope there will be more critical evidence to explain this phenomenon.

## Data Availability

The RNA-seq data was submitted to the NCBI (SRA) database with ID: PRJNA1193114. Further inquiries can be directed to the corresponding author.
