# Supplementary figures and images for: Semaglutide promotes the proliferation and osteogenic differentiation of bone-derived mesenchymal stem cells through activation of the Wnt/LRP5/β-catenin signaling pathway
Source: Front Pharmacol. 2025 Mar 10;16:1539411. doi: 10.3389/fphar.2025.1539411 (PMC11931165; doi:10.3389/fphar.2025.1539411)

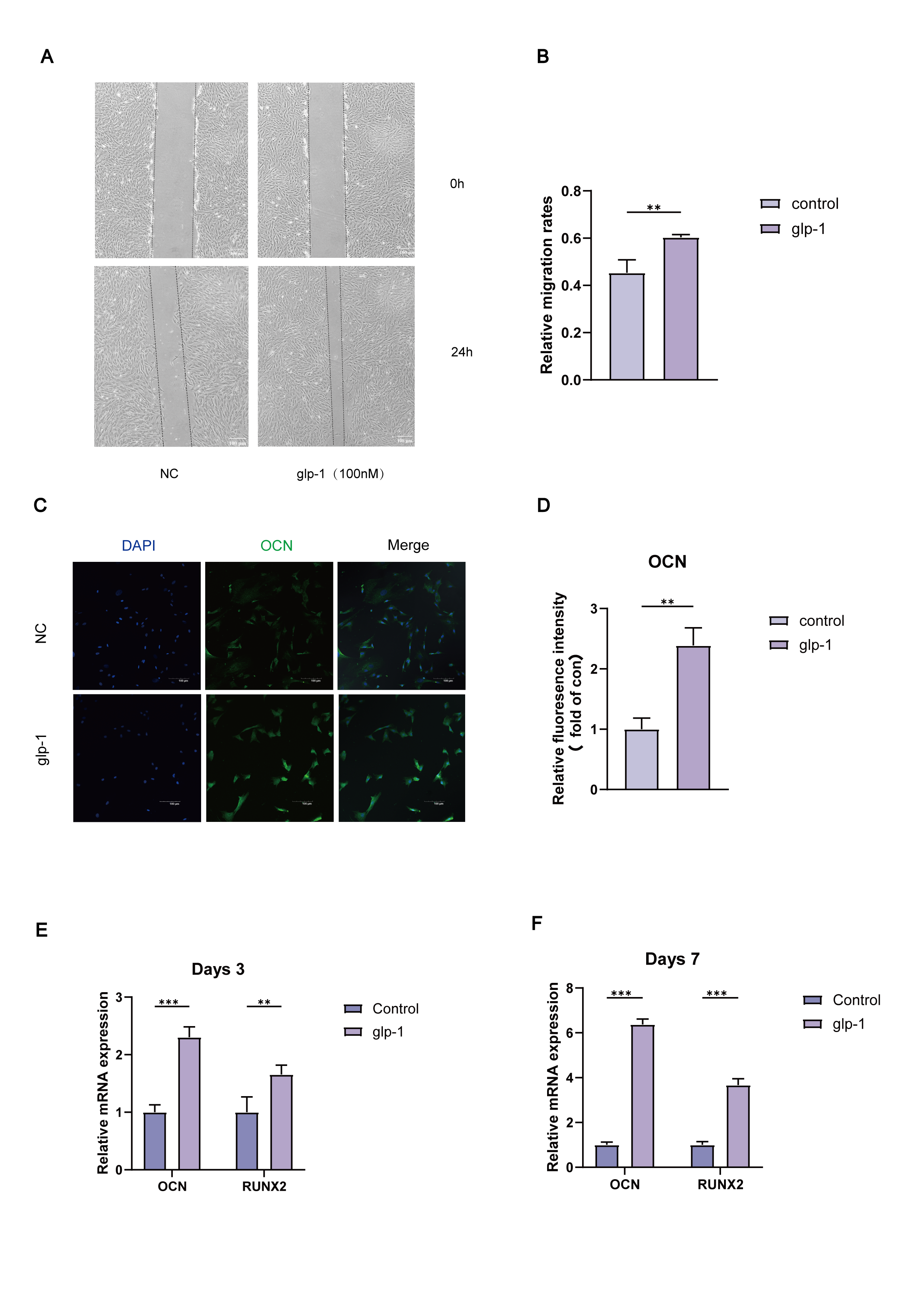

Supplement: Supplementary file 1 [file Image2.tif]

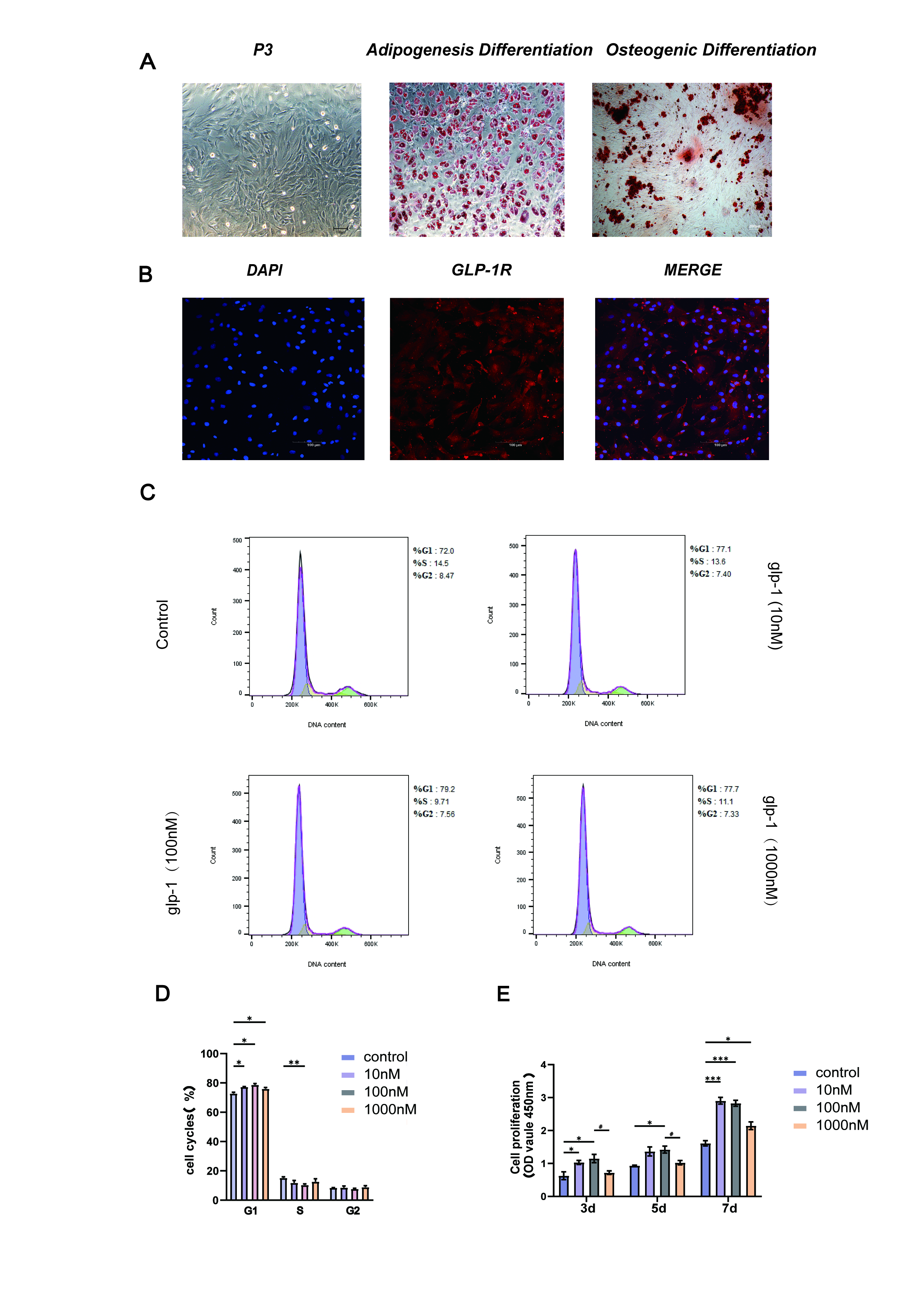

Supplement: Supplementary file 2 [file Image1.tif]
