# Supplementary material for: Semaglutide promotes the proliferation and osteogenic differentiation of bone-derived mesenchymal stem cells through activation of the Wnt/LRP5/β-catenin signaling pathway
Source: Front Pharmacol. 2025 Mar 10;16:1539411. doi: 10.3389/fphar.2025.1539411 (PMC11931165; doi:10.3389/fphar.2025.1539411)
Supplement: Supplementary file 3 [file DataSheet1.docx]

Supplementary Material

# Supplementary Figures


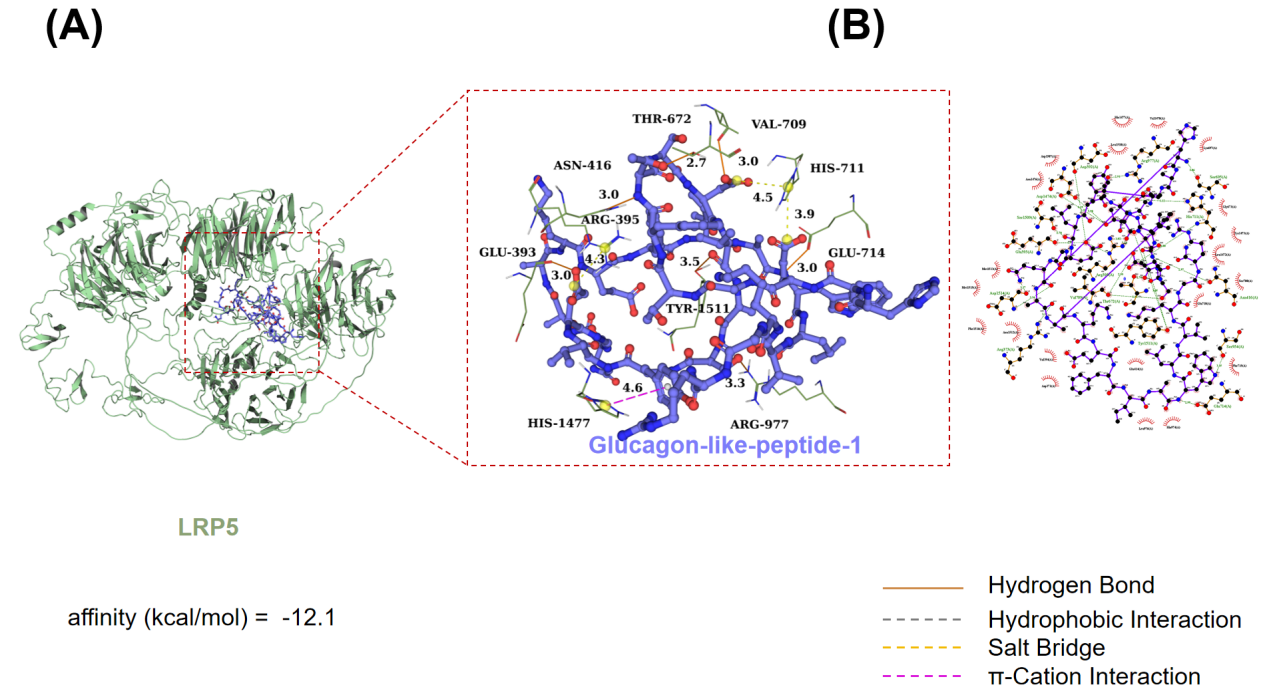


**Supplementary Figure 1.** Binding of Glucagon-like-peptide-1 to its target LRP5 by molecular docking (A) Cartoon representation of a 3D interaction of a small molecule compound and its target (B) a 2D interaction of a compound and its target

# Supplementary Table

**Table 2 up-regulated DEGS in LRP family**

| Gene id | MeanTPM (B) | MeanTPM (A) | log2FoldChange | qValue | result | GeneName |
| --- | --- | --- | --- | --- | --- | --- |
| ENSRNOG00000004221 | 26.67585433 | 3.357430667 | 2.990104964 | 1.80E-09 | up | Lgr5 |
| ENSRNOG00000007765 | 1.618965667 | 0.022954667 | 6.140141098 | 4.22E-08 | up | Frzb |
| ENSRNOG00000008620 | 35.609803 | 10.482394 | 1.764306213 | 9.80E-08 | up | Smad3 |
| ENSRNOG00000013717 | 51.413833 | 7.793170333 | 2.721874317 | 5.46E-05 | up | Bmp6 |
| ENSRNOG00000004517 | 12.384697 | 3.503441333 | 1.82171393 | 0.000174 | up | Igf1 |
| ENSRNOG00000009694 | 15.943374 | 3.583104 | 2.153675147 | 0.000304 | up | Bmp4 |
| ENSRNOG00000015911 | 36.14707333 | 15.43238233 | 1.227918052 | 0.001586 | up | Lrp5 |
| ENSRNOG00000018712 | 0.980855667 | 0.095017 | 3.367783297 | 0.001763 | up | Camk2a |
| ENSRNOG00000009173 | 9.432505 | 2.382224333 | 1.985331682 | 0.002044 | up | Smad6 |
| ENSRNOG00000011451 | 1.138497667 | 0.306200667 | 1.894582006 | 0.002334 | up | Lrp3 |
| ENSRNOG00000016848 | 3.037181667 | 0.696081333 | 2.125405413 | 0.002986 | up | Fzd4 |
| ENSRNOG00000014303 | 24.123107 | 10.17395533 | 1.24553507 | 0.010229 | up | Lrp11 |
| ENSRNOG00000011592 | 156.666725 | 76.618702 | 1.031930303 | 0.019023 | up | Lrp10 |

**Table 3 Wayne diagrams further explain**

| GO.ID | Term | Ontology | Significant | Pvalue |
| --- | --- | --- | --- | --- |
| GO:0033687 | osteoblast proliferation | biological process | 9/2176 | 0.00117 |
| GO:0001649 | osteoblast differentiation | biological process | 35/2176 | 0.00568 |
| GO:0002076 | osteoblast development | biological process | 5/2176 | 0.04096 |
